# Progress in the neurolinguistic assessment in schizophrenia (SZ) – the SchizoLang pilot study

**DOI:** 10.1192/j.eurpsy.2025.2041

**Published:** 2025-08-26

**Authors:** X. Ansorena, S. Mancini, J. Díaz, A. M. Sánchez-Torres, M. J. Cuesta

**Affiliations:** 1 Instituto de Investigación Sanitaria de Navarra (IDISNA), Pamplona; 2 Basque Center on Cognition, Brain and Language, San Sebastian; 3 Servicio de Psiquiatría, Hospital Universitario de Navarra; 4 Departamento de Ciencias de la Salud, Universidad Pública de Navarra, Pamplona, Spain

## Abstract

**Introduction:**

Individuals with SZ show alterations at all levels of language: discourse, lexical-semantics, comprehension and syntax/ morphology. Linguistic capacities are associated with worse occupational, social and quality of life related outcomes (Ehlen et al. Front Psych 2023; 14). Still, language assessment is probably overseen in the cognitive assessment of individuals with SZ

**Objectives:**

1. To review the role of language in the cognitive assessment in SZ
2. To introduce the SchizoLang pilot study

**Methods:**

We reviewed the available cognitive assessment tools in SZ to determine whether language is adequately represented. Consequently, we describe the SchizoLang pilot study

**Results:**

Available instruments for the assessment of cognition in SZ do not adequately evaluate language. Importantly, fluency tests are not representative of language. Table 1 shows the major cognitive assessment tools in SZ.

**
Table 1. Cognition assessment instruments in schizophrenia (adapted from Vita et al. Eur J Psychiat 2022; 65 1-24)**

*The Schizolang pilot study: bridging neurolinguistics and Psychiatry to characterize language in Schizophrenia*

The aims of this pilot study are:

To explore which linguistic domains are altered in people with SZ

To explore the relationship between language and (a) formal thought disorders, (b) psychiatric symptoms, (c) neuropsychological alterations, (d) deficits in psychosocial functioning, and (e) quality of life

*ACS.esp*

ACs.esp is a digital battery for the assessment language in aphasia based on neurolinguistic research. It shows good preliminary validity and reliability (Ansorena et al. 2022; SSTaal, 95 237–240). ACS.esp includes novel measures (see Table 2): an extensive discourse protocol, tasks for sentence planning, and sentence comprehension and production at the syntactic and grammatical levels.

**
Table 1. ACS.esp’s structure with its factors, subfactors, input, stimuli and tasks. Auditory tasks are colored in blue and visual tasks in brown**

ACs.esp is a digital battery for the assessment language in aphasia based on neurolinguistic research. It shows good preliminary validity and reliability (Ansorena et al. 2022; SSTaal, 95 237–240). ACS.esp includes novel measures (see Table 2): an extensive discourse protocol, tasks for sentence planning, and sentence comprehension and production at the syntactic and grammatical levels.

**Image:**

**Figure:**
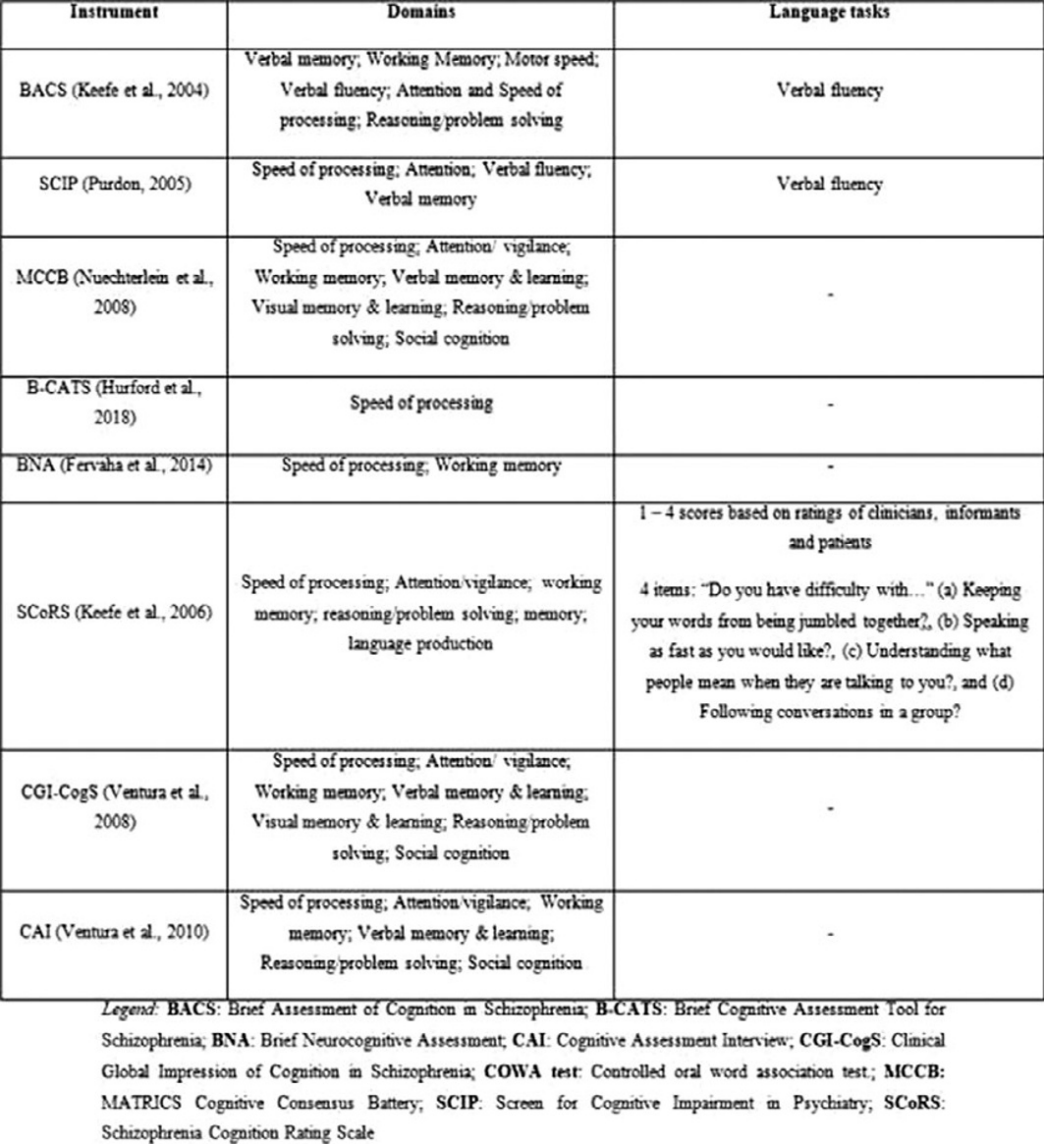

**Image 2:**

**Figure:**
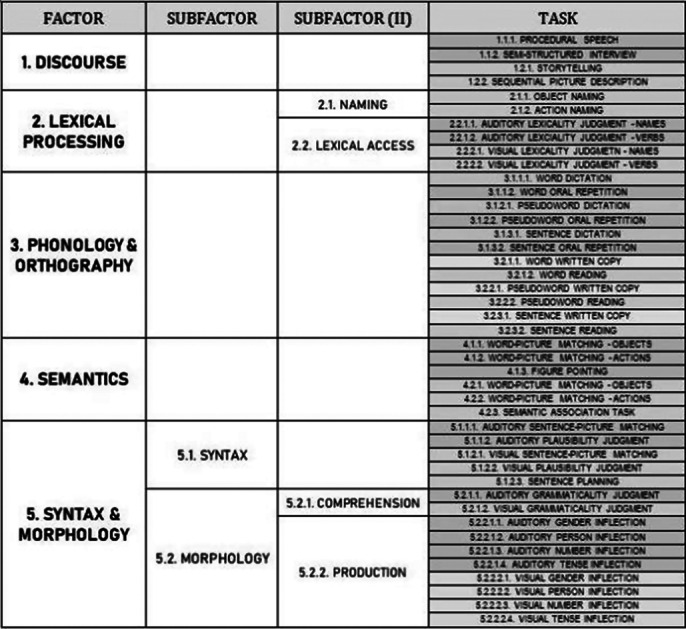

**Conclusions:**

Even if language is altered in SZ, it is not adequately assessed. An extensive characterization of language abnormalities in SZ can guide rehabilitation on communication and functioning; and consequently produce a greater well-being and quality of life. The SchizoLang pilot study will allow establishing a clinician-friendly protocol.

**Disclosure of Interest:**

None Declared

